# Cardiovascular health control in the family health strategy

**DOI:** 10.3389/fcvm.2022.933972

**Published:** 2022-08-18

**Authors:** Gilberto Andrade Tavares, Joathan Borges Ribeiro, Marcos Antonio Almeida-Santos, Antônio Carlos Sobral Sousa, José Augusto Soares Barreto-Filho

**Affiliations:** ^1^Postgraduate Program in Health Sciences, Federal University of Sergipe, Aracaju, Brazil; ^2^Department of Medicine Lagarto, Federal University of Sergipe, Aracaju, Brazil; ^3^Postgraduate Program in Adult Health Nursing, University of São Paulo, São Paulo, Brazil; ^4^Intensive Care Unit, Hospital Sírio-Libanês, São Paulo, Brazil; ^5^Postgraduate Program in Health and Environment, Tiradentes University, Aracaju, Brazil; ^6^São Lucas Clinic and Hospital / Rede D‘Or São Luiz, Aracaju, Brazil; ^7^Division of Cardiology, University Hospital, Federal University of Sergipe, Aracaju, Brazil; ^8^Department of Internal Medicine, Federal University of Sergipe, Aracaju, Brazil

**Keywords:** cardiovascular diseases—epidemiology, community health services [MeSH], family health (source: DeCS BIREMIRE), health policy, health services

## Abstract

**Introduction:**

In Brazil, the Unified Health System (SUS) controls and oversees public health care, and the Family Health Strategy (FHS) is its primary access, with 60% of the population registered in it. The surveillance of risk factors for cardiovascular diseases (CVD) is the responsibility of the FHS. In 2010, the American Heart Association (AHA) proposed the evaluation of seven metrics (smoking, Body Mass Index (BMI), physical activity, diet, total cholesterol, blood pressure and blood glucose) with an aim to monitoring cardiovascular health (CVH). However, the results of the FHS regarding the CVH of the Brazilian population are unascertained.

**Objective:**

Evaluate the control of CVH among adult patients treated by the FHS in the city of Aracaju, Sergipe, Brazil.

**Material and methods:**

A cross-sectional study was conducted using the seven metrics recommended by the AHA to evaluate CVH among patients treated by the FHS. The city of Aracaju has a population of 571,149 inhabitants, with 394,267 > 20 years of age; therefore, it was admitted that in a simple random sample, sampling error of 5% with 95% CI, 329 individuals would be needed.

**Results:**

Among 400 patients, only 32.5% had controlled CVH. In a univariate analysis, the adjusted multivariate analysis found that being female (aOR: 2.07 IC: 1.20 to 3.60 *p*: 0.006) under 45 years old (aOR: 1.61 IC: 1.15 to 2.28 *p*: 0.006) and with the habit of following health advice from family members and neighbors (aOR: 1.28 IC: 1.15 to 2.28 *p*: 0.040) were associated with control of CVH. On the other hand, those ones who had a greater number of children (aOR: 0.91 IC: 0.84 to 0.95 *p*: 0.020) were associated with less control of CVH.

**Conclusions:**

The study showed that only 32.5% of patients have controlled CVH. Being a woman, young and following health advice from family members and neighbors have a positive influence in controlling CVH. More children reduced controlling these metrics.

## Introduction

In 2019, the Unified Health System (SUS), which oversees public health care in Brazil, registered 1,106,916 circulatory system concurrent diseases hospitalizations, including Acute Myocardial Infarction (AMI) and Stroke, being responsible for 9.56% of all hospitalizations (1). The costs of these hospitalizations were R$ 2,550,369,512.25, corresponding to 19.51% of all hospitalization costs in the SUS that year (2). Family Health Strategy (FHS) is the main strategy of primary care of SUS. The FHS is the first element of the continuous care process, addressing the most common problems in the community, providing prevention, treatment and rehabilitation services. In 2019, 60% of Brazilian households were registered in Family Health Units (FHU), which are the health units where the FHS develops upon (3). For the period from 2021 to 2030 regarding CVD, the strategic action plan of primary care has been pursuing the following goals: reduce smoking prevalence by 30%; halt the rise in adult obesity; increase the prevalence of physical activity (PA) practice in free time and also the recommended consumption of fruit and vegetables by 10% (4). However, between August 2018 and December 2020, 45,862,616 complete or partial medical evaluations were performed in the FHUs, a little over 1% (511,046) were related to Cardiovascular Risk Screening, indicating that, despite the magnitude of the problem, screening and monitoring of risk factors for CVD in the FHS has not been assertive (5).

In 2010, the American Heart Association (AHA) proposed to check seven cardiovascular health (CVH) metrics with an auspicious goal of reducing CVD deaths in the U.S. by 20% until 2020. Three of these metrics are considered behavioral (smoking, diet, and PA) and four of these metrics are considered biological [Body Mass Index (BMI), Blood Pressure (BP), Total Cholesterol (TC), and glycemia]. The control of these metrics was stratified into “ideal,” “intermediate,” and “poor” according to AHA (6). Since then, some studies have corroborated the notion in different populations that the greater the number of CVH metrics, the lower the rates of events/mortality due to CVD (7, 8). In Brazil, there are few studies that evaluated the control rate of the seven metrics defined by the AHA in a given population and none of them studied the population treated in the FHS (9, 10). In the Longitudinal Study of Adult Health in Brazil (ELSA-Brazil), comprised of 15,000 employees from public institutions of higher education and research in the Northeast, South, and Southeast regions; on average, the participants of this research obtained only 2.5 ± 1.3 metrics at the ideal level. Only 7.8% of participants had five or more metrics controlled at the ideal level. Of the seven metrics evaluated, ideally controlled smoking (83.5%) was the most prevalent and diet at the ideal level had the worst result (0.8%). Despite the great importance of this study nationwide, the population evaluated in this study does not adequately represent the population that uses the public health service of the country since it evaluated only the employees of those institutions, without covering the other layers of the general population (10).

Considering that the FHS is the best public health program for controlling CVH and used by most of the Brazilian population, we developed this study to verify the control rate of CVH using the seven metrics [smoking, Body Mass Index (BMI), physical activity, diet, total cholesterol, blood pressure and blood glucose] proposed by the AHA in the population treated by the FHS, as well as the stratification frequencies (“ideal,” “intermediate,” and “poor”) and the variables associated with “controlled CVH” using a logistic regression model to check the ones associated with this control. Personal factors that may interfere with the prevention of cardiovascular disease among FHS users will be also observed.

## Materials and methods

This is a cross-sectional study, conducted between August 2018 and December 2020 to evaluate the control of CVH among adult patients treated by the FHS in the city of Aracaju, Sergipe, Brazil. This study followed the STROBE guidelines for reporting observational studies.

For sample calculation, it was considered that Aracaju has 571,149 inhabitants, with 394,267 > 20 years of age; therefore, for a simple random sample, sampling error of 5% with 95% CI, 329 individuals would be required. To ensure the randomness of the sample, a random draw of the FHU was divided into two stages: one random drawing of the neighborhoods and a second one to choose the FHU in these neighborhoods.

The inclusion criteria were adults (aged > 18), residing in the area covered by the FHU for at least 1 year, registered in *e-SUS AB* (SUS Primary Care Registration System) and who had attended a medical appointment within 1 year's time prior to this study. The exclusion criteria were patients who did not meet the inclusion criteria and had allergies, intolerance or alteration of the digestive tract that interfered with food intake, as well as those who presented physical limitations that could prevent or interfere in BP, BMI, and PA performance.

This research was approved by the Research Ethics Committee of the Federal University of Sergipe (CEP/UFS) under 81283617.3.0000.5546. A written informed consent was obtained from all participants. The data sets generated during the present study is stored in a private repository and available for 5 years. The patient is granted the right to request data deletion at any time without any justifiable reason.

### Study variables

The following variables were selected: gender, mean age, age groups (<45 and ≥45 years), marital status (married or unmarried), schooling time in years, schooling (low <12 and high ≥ 12 years), monthly family income (low <420 U.S. Dollars (USD) and average (420–1,050 USD), employment situation (employed or unemployed), health insurance, number of children, number of persons at home, religious attendance and following health advice from family members and neighbors. Housing conditions were also observed: type of construction [masonry home and others (non- and plastered mud-stick house, wood, and reused material)], treated water, sewage, and waste collection. No sex-based or race/ethnicity-based differences were present in this study.

### Metrics collection

In this phase, the seven metrics proposed by the AHA were verified to evaluate CVH, being graded at “ideal,” “intermediate,” and “poor” level. The ideal “level” of each metric defines the “Controlled” CVH in ≥ five metrics at this level, these being as follows (6):

1. Smoking: Never smoked or stopped smoking for more than 12 months.
2. PA practice: ≥150 min/week of PA in moderate intensity or ≥75 min/week in high intensity or ≥150 min/week in moderate and high intensity. To measure the level of PA, the International Physical Activity Questionnaire (IPAQ), short version, validated for the Portuguese (11) was chosen;
3. Diet: Individuals who ingested four to five components of the diet proposed as ideal in the last week, being the components: 4.5 or more portions (>540 g) of fruit or vegetables per day; two or more portions (>200 g) of fish per week; more than 2 >85 g) of whole grains per day; <1,500 mg sodium per day; <1,050 ml of soft drinks per week. Each component equals one point on the scale. The foods were grouped into breads, cereals and tubers; fruit; vegetables, legumes and pulse; eggs, milk and dairy products and meat; pasta and other preparations; sweets and drinks. During the interview, the interviewer wrote down the frequency the subject has that food or drink (Three or more times a day, two or three times a day, once a day, five to six times a week, two to four times a week and one time in the week). To investigate the diet, the Food Frequency Questionnaire (FFQ) of the ELSA-Brazil Study was adopted (12), complemented by the Brazilian Table of Food Composition (11).
4. TC: <200 mg/dl.
5. Glucose: <100 mg/dl.
6. BMI: <25 Kg/m^2^.
7. BP: <120 ×80 mmHg.

The patient was weighed on a digital scale and had his/her height measured with a portable stadiometer, and BMI was calculated (13). Regarding BP, the guidelines decided by the National Center for Health Statistics, sphygmomanometer and cuffs appropriate to arm circumference were respected. In those patients who did not have complementary tests in the year before, laboratory tests were collected with a minimum of 8 h fasting period.

### Data analysis

Exploratory analysis was performed, and continuous variables were described by mean and standard deviation. Categorical variables were described using counts and percentage frequency. The main dependent variable “CVH control” was dichotomized into “Controlled” (≥ five metrics at the ideal level) and “Uncontrolled” (< five metrics at the ideal level). Odds Ratio (OR) was estimated and adjusted by Poisson regression with standard errors with 95% CI for “Controlled” CVH. Pearson Chi-Square and Mann-Whitney tests were used to evaluate the differences between the groups, as appropriate. The hypothesis of adherence of the continuous variables to the normal distribution was evaluated by the Shapiro-Wilks test. Associations of CVH metrics and study variables were analyzed using univariate logistic regression analyses. For the adjusted model, the Backward method was used with inclusion criteria of the variables: univariate significance below 0.2 and permanence criteria of significance lower than 0.05. All tests were two-tailed, and the significance level was set at p <0.05. The software used was R Core Team 2021 (Version 4.05).

## Results

### Characteristics of patients

**Figure 1:**
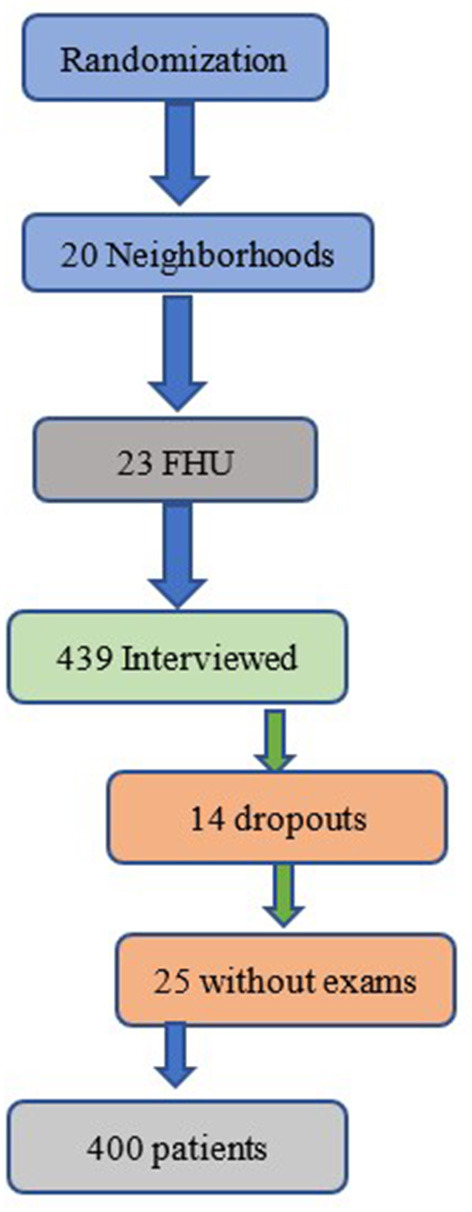
Randomization number of neighborhoods, FHU, excluded (abandonments and without complete examinations) and the final sample of patients.

Data were collected in 20 of the 39 neighborhoods of the city, and 23 FHUs were visited. We interviewed 439 patients, however, 14 of them did not complete the interview and 25 did not perform all the necessary complementary tests. Thus, the final sample consisted of 400 patients (Figure 1).

The mean age of the patients was 45.1 ± 15.5 years, female (85%) and unmarried (61%). Most of them had low and medium schooling (66.5%), low monthly family income (75.3%), unemployed (63.3%) and no access to supplementary health (91.5%). Regarding religious behavior, the majority attended some religious service (74.5%). Approximately half of the patients reported that they followed health advice from family members and neighbors. Average number of children 2.3 (±1.9) and 3.4 (±1.6) people per household. The vast majority lived in brickwork house (98.8%), had also access to treated water (98.3%), which is often associated with sanitary sewage (89.3%) and regular waste collection (97.5%) (Table 1).

**Table 1:** Population characteristics (*n* = 400).

**Sociodemographic characteristics**	
	**Total** ***n** **=*** **400**
**Age**, mean (SD)	45.1 (15.5)
**Age groups** ***, n (%)***	
≥45	201 (50.2)
<45	199 (49.8)
**Gender**, ***n*** **(%)**	
Female	340 (85)
Male	40 (15)
**Marital status**, ***n*** **(%)**	
Unmarried	244 (61)
Married	156 (29)
**Study time in years** *, mean (SD)*	9.3 (3.5)
**Schooling**, ***n*** **(%)**	
Low and medium	266 (66.5)
High	134 (33.5)
**Monthly family income**, ***n*** **(%)**	
Low	301 (75.3)
Average	99 (24.7)
**Employment status**, ***n*** **(%)**	
Unemployed	253 (63.3)
Employee	147 (37.7)
**Health convention**, ***n*** **(%)**	
No	366 (91.5)
Yes	37 (8.5)
**Attendance of religious space**, ***n*** **(%)**	
No	102 (25.5)
Yes	298 (74.5)
**Follow health advice from family members/neighbors**, ***n*** **(%)**	
No	188 (47)
Yes	212 (53)
**Number of children**, mean (SD)	2.3 (1.9)
**Number of people at home**, mean (SD)	3.4 (1.6)
**Type of construction**, ***n*** **(%)**	
Masonry home	395 (98.8)
Other	5 (1.2)
**Treated water**, ***n*** **(%)**	
No	7 (1.7)
Yes	393 (98.3)
**Sanitation**, ***n*** **(%)**	
No	43 (10.7)
Yes	357 (89.3)
**Waste collection**, ***n*** **(%)**	
No	10 (2.5)
Yes	390 (97.5)

*n—absolute frequency. %—percentage relative frequency. DP—Standard Deviation*.

### Evaluation metrics in CVH

**Figure 2:**
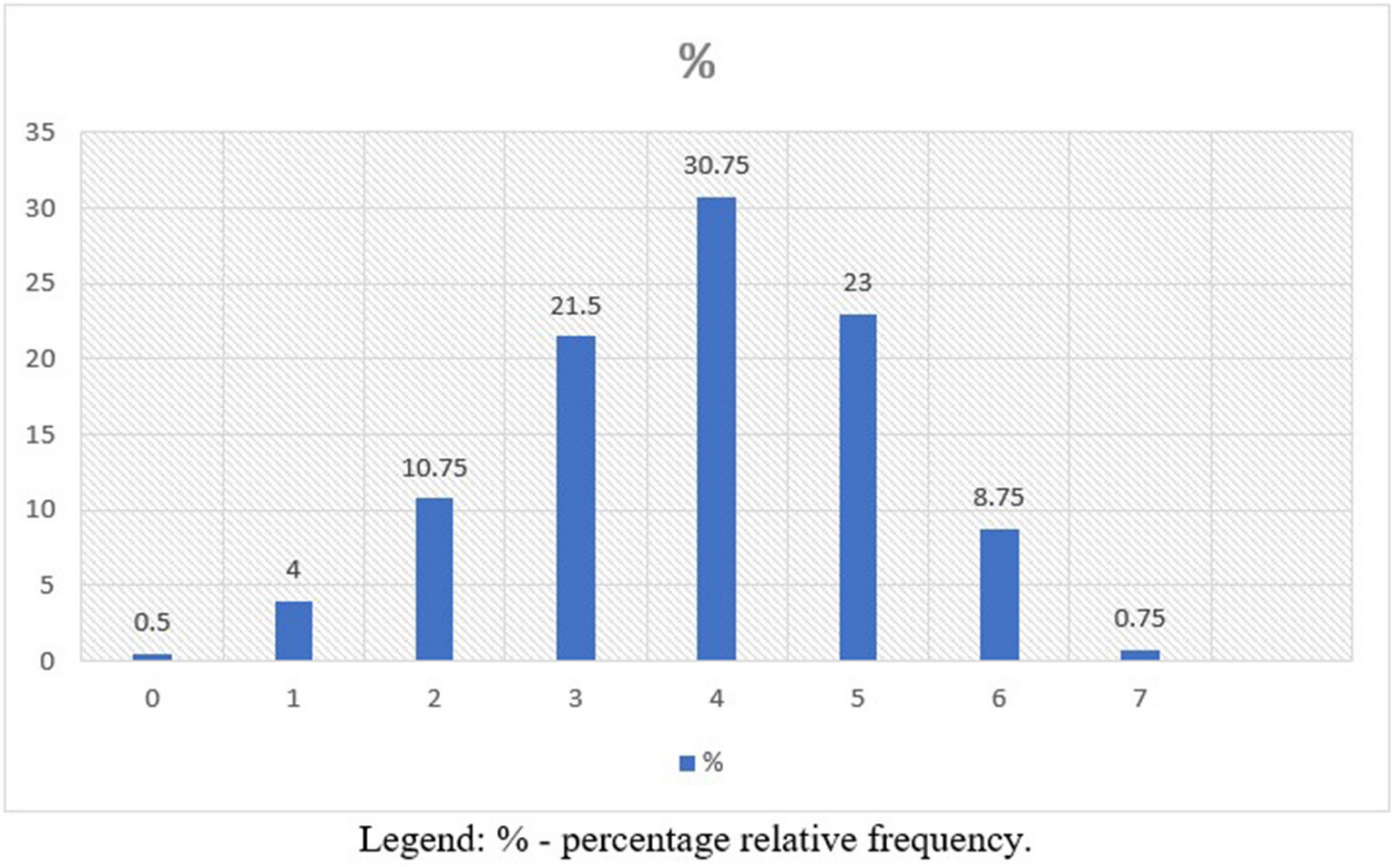
Number of ideal CVH metrics in the population (*n* = 400).

In our study, only 130 patients (32.5%) had “Controlled” CVH. It was observed that only three patients (0.75%) achieved all seven controlled metrics at the ideal level. The majority of the population (62.75%) presented two to four controlled metrics at the ideal level. Only 16 interviewees (4%) got only one ideal metric in CVH, and two other ones (0.5%) did not score (Figure 2). Evaluating the control of each metric, Smoking reached 92%, glycemia 79.3%, PA 70.8% and CT 66.5% at “ideal” levels. BP at the “ideal” level was found in 35% of patients.

### Dichotomization of metrics in “controlled” and “uncontrolled” CVH

The univariate analysis of the dichotomization of the metrics in “Controlled” and “Uncontrolled” CVH showed that: being <45 years of age, having longer schooling time, high schooling and fewer children were associated with “Controlled” CVH (*p* <0.001). Being female had also a good association (*p* = 0.005) with “Controlled” CVH (Table 2).

**Table 2:** Dichotomization of the classification “Controlled” (≥5 ideal metrics in CVH) and “Uncontrolled” (<5 ideal CVH metrics) by variables (*n* = 400).

	**Patients (*n =* 400)**		
**Variable**	**“Controlled” (*n =* 130)**	**“Uncontrolled” (*n =* 270)**	***p*-value**
**Age in years** *, mean (SD)*	37 (14.8)	49 (14.3)	<0.001 ^M^
**Age group in years** ***, n (%)***			
≥45	32 (24.6)	169 (62.6)	<0.001 ^Q^
<45	98 (75.4)	101 (37.4)	
**Gender** ***, n (%)***			
Female	120 (92.3)	220 (81.5)	0.005 ^Q^
Male	10 (7.7)	50 (18.5)	
**Marital status** ***, n (%)***			
Not married	82 (63.1)	162 (60)	0.555 ^Q^
Married	48 (36.9)	108 (40)	
**Time study in years** *, mean (SD)*	10.3 (3)	8.8 (3.6)	<0.001 ^M^
**Schooling** ***, n (%)***			
Low and medium	70 (53.8)	196 (72.6)	<0.001 ^Q^
High	60 (46.2)	74 (27.4)	
**Monthly family income** ***, n (%)***			
Low	96 (73.8)	205 (75.9)	0.652 ^Q^
Average	34 (26.2)	65 (24.1)	
**Employment status**, ***n (%)***			
Unemployed	81 (62.3)	172 (63.7)	0.786 ^Q^
Employee	49 (37.7)	98 (36.3)	
**Health convention**, ***n (%)***			
No	121 (93.1)	245 (90.7)	0.433 ^Q^
Yes	9 (6.9)	25 (9.3)	
**Religious attendance**, ***n (%)***			
No	31 (23.8)	71 (26.3)	0.598 ^Q^
Yes	99 (76.2)	199 (73.7)	
**Agree guidelines**, ***n*** **(%)**			
No	52 (40)	136 (50.4)	0.052 ^Q^
Yes	78 (60)	134 (49.6)	
**Number of children** *, mean (SD)*	1,6 (1.3)	2.6 (2)	<0.001 ^M^
**Number of persons** *, average (SD)*	3,4 (1.5)	3.4 (1.7)	0.435 ^M^
**Type of construction**, *n (%)*			
No	2 (1.5)	3 (1.1)	0.719 ^Q^
Yes	128 (98.5)	267 (98.9)	
**Treated water** ***, n (%)***			
No	1 (0.8)	6 (2.2)	0.299 ^Q^
Yes	129 (99.2)	264 (97.8)	
**Sanitation** ***, n (%)***			
No	14 (10.8)	29 (10.7)	0.993 ^Q^
Yes	116 (89.2)	241 (89.3)	
**Waste collection** ***, n (%)***			
No	3 (2.3)	7 (2.6)	0.864 ^Q^
Yes	127 (97.7)	263 (97.4)	

*n—absolute frequency. %—relative percentage frequency. DP—Standard Deviation*.

Q *–Pearson Chi-Square Test*.

M *–Mann-Whitney test*.

In the multivariate analysis, after adjustments, being younger (<45 years of age) implies a chance of having 61% “Controlled” CVH. Females (107%) and following health advice from family members and neighbors (28%) are also associated with “Controlled” CVH. Having a higher number of children reduces the chance of having “Controlled” CVH by up to 9% (Table 3).

**Table 3:** Associations between “Control” CVH (>5 metrics at the ideal level) by search variables (*n* = 130).

	**“Controlled CVH”**		
**Variables**	**OR (IC 95%)**	**aOR (IC 95%)**	***p*-value**
Age group <45 years	3.09 (2.18–4.38)	1.61 (1.15–2.28)	0.006
Female	2.12 (1.18–3.80)	2.07 (1.20–3.60)	0.009
Low and middle schooling	1.70 (1.29–2.24)		
Share family/neighbor decisions	1.33 (0.99–1.78)	1.28 (1.15–2.28)	0.040
Largest number of children	0.78 (0.71–0.86)	0.91 (0.84–0.95)	0.020

*n—absolute frequency. %—relative percentage frequency. OR, Odds Ratio; aOR, Adjusted Odds Ratio; IC95%, Interval with 95% confidence*.

## Discussion

This observational, cross-sectional study, evaluating the control of CVH in patients treated by the FSH, we demonstrated that, firstly, only 1/3 of the patients had controlled CVH; secondly, smoking (92%) was the metric with the highest prevalence at the ideal level, followed by blood glucose (79.3%), PA (70.8%) and TC (66.5%); thirdly, the ideal level metrics with the lowest prevalence were BP (35%), BMI (30.8%), and diet (10.8%); fourthly, the adjusted multivariate analysis showed that being under 45 years of age, female and sharing decisions about their health with neighbors and family members are associated with “Controlled” CVH. Having more children reduces this association. This study is the first one that evaluated all seven CVH metrics proposed by the AHA in the FSH, showing that this control is not adequate in the treated population.

There is no similar study about the association of CVH metrics of AHA and the FSH in Brazil, but there are associations among “Controlled” CVH and reduction of cardiovascular events and mortality (7, 14) worldwide. In a Finnish cohort, men with “Controlled” CVH showed an 85% reduction in sudden death compared to men with < two metrics at the ideal level in CVH (15). Similar findings were shown in Australia, where patients with Controlled CVH showed 66% reduction in CVD risk (ORa 0.34; CI 95% 0.22 to 0.54). The addition of each metric at the “ideal” level, there was a 21% reduction in CVD risk (ORa: 0.79; 95% CI 0.73 to 0.84) after adjustments (16).

Smoking at the “ideal” level presented the best results in our study, similar to the worldwide trend of tobacco reduction, where 20.5% of the population is expected to be smokers in 2025 (20). In the sample, 80% of the volunteers had blood glucose at the ideal level. This can be explained by the fact that most patients are young adults. Unfortunately, in the last 29 years, there has been an increase in high blood glucose diagnoses from 7.88 to 11.72%, resulting in 6.23 million deaths in 2019 worldwide (21). In our study, 71% of patients were also physically active. Our interviewees had a gain in work-related activity and household activities and, although leisure-time physical activity has increased in recent years. But trends in the use of technologies, especially among younger people, can influence the growth of sedentary lifestyle (17). Cholesterol at the ideal level was found in 66.5% of patients. Again, because it is a younger population, these numbers are expected. In a recent meta-analysis of the prevalence of NCDs, a prevalence of 51.2% of Dyslipidemia (23) was verified. Among other measures, the consumption of whole grains, fish and food that is not or little processed can help reduce cholesterol levels (18).

Blood pressure at the ideal level was found in only 35% of patients. High blood pressure is one of the main risk factors for CVD, leading to premature mortality in a young and full productive population. According to the NHANES, ~46% of the adult population of the USA is hypertensive (17), reaping this economically active population from their families and society. Another metric with low ideal level was BMI, where only 30.8% of the interviewees reached this level. Obesity is one of the main public health problems, with an increasing number in Brazil and worldwide, and is currently considered a pandemic by the WHO (19). These two metrics are intrinsically connected to diet that, in our study, reached at an “ideal” level of control just over 10% of the patients, similar to other studies worldwide (8, 20, 21). This can be modified, as demonstrated by a recent literature review on the engagement and participation in the promotion of these metrics in the black American community, where 16 of the 23 studies obtained results of changing the dietary pattern, increasing the intake of fruit, vegetables, whole grains, fibers and, at the same time, reducing the consumption of beverages and fat (22). In Brazil, the food guide of the Brazilian population, which pursues the choice of fresh or minimally processed foods and culinary preparations instead of ultra-processed foods, can be a guide in improving the population's diet. A recent study, which described the adequacy of eating habits in the regions of Brazil according to the guidelines of this guide is associated with a better food adequacy and that the dissemination of these guidelines should be a goal of the Brazilian Ministry of Health due to their potential in improving the diet of the population (23).

In our study, younger women were more likely to have “Controlled” CVH. This finding is in line with other studies. In a cross-sectional study in Bosnia, 54% of respondents were women, with a higher number of metrics at the “ideal” level. In the same study, it was also observed that being younger (*p* = 0.001) was also associated with “Controlled” CVH (24). In study Paris Prospective Study 3 (PPS3), where the majority of the population studied was male and although females were slightly older, with lower educational level, more fragile and depressed, had better “ideal” level indexes for most metrics, except CT and PA (25).

It was seen that sharing with neighbors and family members decisions about their health was associated with “Controlled” CVH. In a systematic review, observing 47 studies that addressed care centered on the person and the family, among other variables, it was verified that the improvement of the clinical-family therapeutic alliance led to the reduction of re-hospitalizations of patients and changes in the health of family members (26). In a recent article addressing the environmental determinants of CVR, it was found that when it comes to obesity, if one sibling becomes obese, the chances of the other becoming obese increase by 40%. Therefore, it was concluded that families may have a similar risk of CVD not only because they share similar genes, but also because they share similar life habits (27).

A greater number of children was related to a sub-optimal control of CVH. This can be explained by the fact that the more people living in a household, the lower the family income, thus leading to less access to food and an unhealthier lifestyle. We found in the literature indirect evidence about this finding. In a study that evaluated central adiposity in a population attended by SUS, in the countryside of Bahia, Brazil, in females, not having children represented a reduction of 72% (OR 0.28; 95% CI: 0.19 to 0.43) at risk of central adiposity (28). In another study that evaluated the incidence of DM among elderly caregivers treated by the FHS, it was observed, among other factors, that a higher number of people in the household represented a 25% chance of developing the disease in the multivariate analysis (OR: 1.25; 95% CI: 1.03 to 1.52) (29). Finally, in a study in Florianópolis, a southern Brazilian city, evaluating socioeconomic inequalities in quality of life among adults and chronic diseases, it was found that individuals over 40 years of age or with lower family income had a low score in the psychological domain of Quality of Life and this interfered directly in the control of those chronic diseases (30).

The strengths of this study are the variability of the neighborhoods and FHU surveyed. A representative sample of the population treated in the FHS was also designed. However, some limitations of this study must nonetheless be addressed. Firstly, the results are based on a sample of that population and therefore cannot be generalized to other population groups. Secondly, it is a cross-sectional study, and we were unable to examine the casualty between the metrics in CVH and the performance of the FHS in their modification. Thirdly, given the follow-up period and the use of baseline assessments, CVH metrics levels are likely to change over time. This may be due to some potential factors such as aging, disease, lifestyle changes and use of lipid and antihypertensive drugs, leading to underestimation of true associations as a result of regression dilution bias. Fourthly, some variables, such as smoking, PA and diet were self-reported and, therefore, are subject to miscommunication. Finally, the majority of the interviewees were female and, therefore, the male population may be underrepresented. We can see in the literature that this is a constancy in most surveys within the FHS (31). In a study evaluating FSH in the PNS with 7,213 patients, only 38% were male (32).

The study showed that only 1/3 of patients have controlled CVH. In the population studied, smoking was highlighted as the best ideal metric in CVH, followed by blood glucose, physical activity and TC. Otherwise, BP, BMI, and diet presented lower ideal control rates. Successful prevention efforts that improve healthy diet and physical activity should result in an improvement in BMI and BP, and even modest changes may result in substantial cardiovascular benefits. Being a woman, young and following health advice from family members and neighbors had a positive influence in controlling CVH. More children reduced control of these metrics. Our data suggest that the control of CVH factors of FSH patients is sub-optimal and that new public health policies should be implemented to improve the control of CVH in the FSH.

## Data availability statement

The raw data supporting the conclusions of this article will be made available by the authors, without undue reservation.

## Ethics statement

The studies involving human participants were reviewed and approved by Research Ethics Committee of the Federal University of Sergipe (CEP/UFS). The patients/participants provided their written informed consent to participate in this study.

## Author contributions

GT contributed to the conception, design, acquisition, analysis, interpretation of data, elaboration, important critical review, and final approval of the version to be published. To sum up, he was responsible for handling all aspects, ensuring that issues related to the accuracy or integrity of any part of the work could be properly investigated and solved. JB-F contributed to the conception, design, acquisition, analysis, data interpretation, elaboration, important critical review, and final approval of the version to be published. JR contributed to the conception, design, acquisition, analysis, interpretation of data, preparation, and critical review of the version to be published. AS and MA-S contributed to the interpretation of data, preparation, and critical review of the version to be published. All authors contributed to the article and approved the submitted version.

## Conflict of interest

Authors MA-S, AS, and JB-F were employed by Rede D'Or São Luiz. The remaining authors declare that the research was conducted in the absence of any commercial or financial relationships that could be construed as a potential conflict of interest.

## Publisher's note

All claims expressed in this article are solely those of the authors and do not necessarily represent those of their affiliated organizations, or those of the publisher, the editors and the reviewers. Any product that may be evaluated in this article, or claim that may be made by its manufacturer, is not guaranteed or endorsed by the publisher.
